# Correlation Between Microbial Diversity and Volatile Flavor Compounds of *Suan zuo rou*, a Fermented Meat Product From Guizhou, China

**DOI:** 10.3389/fmicb.2021.736525

**Published:** 2021-10-20

**Authors:** Hanyu Wang, Wei Su, Yingchun Mu, Chi Zhao

**Affiliations:** ^1^School of Liquor and Food Engineering, Guizhou University, Guiyang, China; ^2^Guizhou Key Laboratory for Storage and Processing of Agricultural and Animal Products, Guizhou University, Guiyang, China; ^3^Animal Disease Control and Prevention Center of Guizhou Province, Guizhou University, Guiyang, China

**Keywords:** fermented meat, microbial diversity, volatile flavor compounds, physicochemical properties, correlation

## Abstract

*Suan zuo rou* (*SZR*), a traditional fermented meat from Guizhou province, China, is loved by local people for its unique flavor and nutritional value. However, the microbial communities and related flavor characteristics of *SZR* from different regions of Guizhou are unclear. We studied the correlation between the microbial communities and the physicochemical properties and volatile flavor compounds (VFCs) of 15 *SZR* samples from three regions in Guizhou province. The microbial community structure of *SZR* was determined by high-throughput sequencing and VFCs were identified by headspace-solid phase microextraction combined with gas chromatography-mass spectrometry. The results indicated that the microbial communities of *SZR* varied among the regions, as evidenced by the relative abundance of *Weissella*, *Staphylococcus*, *Brochothrix*, *Kazachstania*, and *Debaryomces*. There were also significant differences in pH, water activity, NaCl, and total volatile basic nitrogen (*P* < 0.05). Based on orthogonal projections to latent structures and Pearson’s correlation coefficient, we showed that *Wickerhamomyces*, *Kazachstania*, *Lactobacillus*, *Weissella*, *Brochothrix*, *Debaryomyces*, *Staphylococcus*, *Pediococcus*, *Pichia*, *Candida*, and *Leuconostoc* were highly correlated with 48 VFCs (| ρ| > 0.8, *P* < 0.05). Redundancy analysis showed that most of the dominant bacteria were positively related to water activity, whereas *Lactobacillus* was positively related with pH, and negatively related with total volatile basic nitrogen.

## Introduction

Since ancient times, meat products have played an important role in the human diet, providing us with sufficient protein and energy (Mourente and Bell, 2006). Fermentation, a traditional processing technology, is used to preserve food ingredients to extend shelf life and enhance nutritional value, as well as promote the development of the texture and flavor of foods (Liu and Zhou, 2011).

Fermented foods are affected by more complex factors and mechanisms than other types of food, and a wide variety of microorganisms that occur during natural fermentation are responsible for giving cured meat its unique flavor. Li et al. (2019) reported that the unique flavor of fermented meat products comes from a variety of microorganisms harvested during the natural fermentation process, with enzymes in the raw meat closely related to the flavor. Microorganisms are involved in lipid hydrolysis and autoxidation, and hydrolysis of proteins and carbohydrates, which have an impact on flavor formation (Sidira et al., 2016). Microbial catabolism of raw materials is also closely related to the formation of volatile flavor compounds (VFCs) in fermented foods (Yao et al., 2020).

*Suan zuo rou* (*SZR*) is a traditional natural fermented meat from Guizhou province, with a longer shelf life than fresh meat products. The traditional fermentation process of *SZR* is based on fresh pork, which is sealed in a tank with salt and rice noodles for 1–2 months to ferment under anaerobic conditions. The end product is favored because of its unique flavor, nutritional value, and non-greasy characteristics. Lv et al. (2019b) showed that *Lactobacillus* played a dominant role at the end of fermentation and could inhibit the growth of species with poor acid resistance and spoilage microbes. Several researches mainly focus on the isolation and identification of bacteria, and sensory quality (Chen et al., 2014, 2020; Hu et al., 2017). Another study reporting the natural fermentation process of sour meat has showed that *L. plantarum* may be regarded as an important potential indicator of fermentation maturation and quality (Lv et al., 2019a). The process of *SZR* production, environmental conditions, and regional differences; however, it can lead to different types and metabolic characteristics of microbial flora, and there may be significant differences in the quality and stability of *SZR*. Therefore, it is important to understand the correlation between the microbiota and flavor of *SZR*, and in particular, the relationship between core microorganisms and characteristic flavors. In the past few years, high-throughput sequencing (HTS) has been widely employed in food microbiology, as it offers insights beyond the limitations of traditional culture methods (Zheng et al., 2018). A number of studies have been conducted on the correlation between microbial community and flavor in fermented foods such as sausages (Quijada et al., 2018), Jinhua ham (Wang et al., 2021), traditional white sour soup (Liu et al., 2020), and Pao cai (Kim et al., 2014). However, few studies have yet been carried out for *SZR*.

In this study, the physicochemical properties, bacterial and fungal community structures, and VFCs in *SZR* were analyzed using fermentation feature testing, HTS technology, and headspace-solid-phase microextraction (HS-SPME) combined with gas chromatography-mass spectrometry (GC-MS) analysis, respectively. Furthermore, the redundancy analysis (RDA) and Pearson’s correlation coefficient analysis were used to assess the interrelationships between microbial community and physicochemical properties and VFCs in *SZR*, with the aim of providing a theoretical basis for the standardization of production, food quality, and safety control.

## Materials and Methods

### Sample Collection

In total, 15 *SZR* samples were sampled from three different regions in Guizhou province, China, including Zunyi (ZY), Meitan (MT), and Libo (LB). The primary ingredients and processing conditions of samples are listed in Supplementary Table 1. Each region of *SZR* was obtained from a local company in five separate lots. Each sample collected weighed 500 g. All *SZR* was produced using a traditional natural fermentation method, fresh pork (purchased in the local market, respectively), washed and sliced, kneaded and decorated with 5% salt and 10% rice flour, plugged the jar mouth with fresh brown leaves, inverted, sealed and naturally fermented for 2 months. And the production process and basic recipe were similar, but with some differing ingredients such as the amount and type of spices. Completely fermented samples were collected, and stored at −80°C until use.

### Physiochemical Properties Determination

The pH of the *SZR* was measured according to the method described by Berardo et al. (2016), using a pre-calibrated pH meter (Sabxin SX-620, Shanghai, China) at room temperature. Water activity (a_w_) was measured with a water activity meter (Five Easy Plus FE28, Mettler Toledo International Inc., Columbus, OH, United States).

The NaCl content of the samples was tested according to the Chinese national standard (GB5009.44-2016). In brief, 10 g of each sample was added to 50 mL of water at 70°C and boiled for 15 min. After ultrasonic treatment for 20 min, 2 mL of precipitators I and II was added successively [1 mol/L K_4_Fe(CN)_6_⋅3H_2_O and Zn(CH_3_CO_2_)_2_, respectively; Sigma-Aldrich Co., St Louis, MO, United States]. After being left to stand for 30 min, 50 mL of filtrate was extracted. Using 1 mL of K_2_Cr_2_O_7_ (10%, Shanghai Aladdin Biochemical Technology Co., Ltd., Shanghai, China) as an indicator, the standard titration solution of AgNO_3_ (0.02 mol/L, Sigma-Aldrich Co.) was titrated for quantification.

The total volatile basic nitrogen (TVB-N) was determined by the semi-micro nitrogen determination method and defined as mg/100 g. In brief, 100 mL of distilled water was added to each 10 g sample, which was shaken and then left to infuse for 30 min. The solution was filtered before use. The receiving agent consisted of 10 mL of 2% H_3_BO_3_ w/v (Aladdin Co., Ltd.) and mixed indicator (1 g/L methyl red absolute ethanol:1 g/L bromocresol green absolute ethanol in a 1:5 ratio; Macklin Inc., Shanghai, China) This was placed at the lower end of the condensing pipe; 5 mL of filtrate and 5 mL of 1% MgO were then placed in the reaction chamber, which was quickly plugged, and the sample was steam distilled for 5 min. The assay was titrated with 0.01 M HCl (Shanghai Hutian Chemical Co., Ltd., Shanghai, China).

### DNA Extraction and Polymerase Chain Reaction Amplification

Total genomic DNA was isolated from samples using the DNeasy PowerSoil Pro Kit (Qiagen, Venlo, Netherlands) according to the manufacturer’s protocol. DNA quality and quantity were assessed using absorbance ratios of 260–280 nm and 260–230 nm. The DNA was then stored at −80°C until further processing. The V3-V4 region of the bacterial 16S rRNA gene was amplified with primers 338F (5′-ACTCCTACGGGAGGCAGCAG-3′) and 806R (5′-GGACTACHVGGGTWTCTAAT-3′) (Jiang et al., 2020). The ITS1 region of the fungi was amplified with the forward primer ITS1F (5′- CTTGGTCATTTAGAGGAAGTAA-3′) and the reverse primer ITS1R (5′-GCTGCGTTCTTCATCGAT GC-3′) (Buée et al., 2010).

Polymerase chain reaction (PCR) amplification was performed using a total volume of 50 μL, which contained 10 μL of buffer, 0.2 μL of Q5^®^ High-Fidelity DNA Polymerase (New England BioLabs Inc., Ipswich, MA, United States), 10 μL High GC Enhancer (New England BioLabs), 1 μL of dNTP, 10 μM of each primer, and 60 ng of genomic DNA. Thermal cycling conditions were as follows: an initial denaturation at 95°C for 5 min, followed by 15 cycles at 95°C for 1 min, 50°C for 1 min, and 72°C for 1 min, with a final extension at 72°C for 7 min. The PCR products from the first step of PCR were purified using VAHTS DNA Clean Beads (Vazyme Biotech Co., Ltd., Nanjing, Jiangsu, China). A second-round of PCR was then performed using a total volume of 40 μL that contained 20 μL of 2 × Phusion High-Fidelity Master Mix (New England BioLabs), 8 μL of ddH_2_O, 10 μM of each primer, and 10 μL of PCR products from the first step. Thermal cycling conditions were as follows: an initial denaturation at 98°C for 30 s, followed by 10 cycles at 98°C for 10 s, 65°C for 30 s, and 72°C for 30 s, with a final extension at 72°C for 5 min. Finally, all PCR products were quantified using a Nanodrop^TM^ 2000 spectrophotometer (Thermo Scientific, Wilmington, DE, United States) and pooled together. HTS analysis of bacterial rRNA and fungal ITS1 genes was performed on the purified, pooled sample using the Illumina Hiseq 2500 system (San Diego, CA, United States) (2 × 250 paired ends) at Biomarker Technologies Corporation, Beijing, China.

### High-Throughput Sequencing and Sequence Analysis

Raw FASTQ sequencing files obtained from the Illumina platform were quality-filtered with Trimmomatic software (version 0.33) (Bolger et al., 2014) and merged using FLASH software (version 1.2.7) (Magoc and Salzberg, 2011). The USEARCH software (version 11.0^1^) was used to cluster data at a 97% similarity level to obtain operational taxonomic units (OTUs) (Bokulich et al., 2013). OTUs were then taxonomically annotated based on Silva^2^ taxonomic databases, setting the comparison threshold at 80%. Lastly, QIIME software (version 4.2) (Edgar et al., 2011) was used to identify and remove the chimeric genes to get the high-quality tag sequences (Caporaso et al., 2010). The alpha diversity indexes, Shannon index curves, and rarefaction curves were evaluated using MOTHUR software (version 1.30) and R software (version 3.6.3^3^) (R Development Core Team, 2009), and beta diversity analysis, principal coordinates analysis (PCoA), and non-metric multidimensional scaling (NMDS) were conducted using QIIME software (Caporaso et al., 2010). To establish the co-occurrence networks of core bacterial and fungal genera, we calculated Pearson’s correlation coefficients using R software with the ‘‘corrplot’’ package and applied Gephi software (version 0.9.^4^) for visualization.

### Analysis of Volatile Compounds

A TRACE^TM^ 1300 gas chromatograph coupled to a TSQ^TM^ 8000 Evo mass spectrometer (GC-MS, Thermo Electron Corp., Waltham, MA, United States) equipped with a DB-5MS capillary column (30 m length × 0.25 mm inner diameter × 0.25 μm film thickness; Agilent Inc., Santa Clara, CA, United States) and a flame ionization detector (Agilent Inc.) was used for the detection of VFCs in *SZR*, using headspace-solid-phase microextraction-gas chromatography-mass spectrometry (HS-SPME-GC-MS). We used the methods described in Marušić et al. (2014) with modifications. Briefly, each sample (2.5 g) was minced and packed into a 20 mL headspace flask along with a saturated saline mixture (7 mL) and cyclohexanone (20 μL, 20 μg/mL), and then equilibrated in a water bath at 60°C for 20 min. The SPME (50/30 μm DVB/Carboxen/PDMS, Supelco, United States) was exposed in the headspace of the vial at 40°C for 180 min to adsorb VFCs. Then, the fiber was immediately inserted into the GC injector port and heated at 230°C for 5 min. Helium (purity: 99.999%) was used as the carrier gas for GC at a flow rate of 1 mL/min and no shunt mode, with an oven temperature program as follows: initial temperature 40°C for 5 min, increased at a rate of 5°C/min to 150°C, 150°C for 3 min, then increased to 240°C at a rate of 5°C/min and finally 240°C for 5 min. During operation of the mass spectrometer, electron ionization was maintained at 70 eV, ion source temperature at 230°C, and transmission line temperature at 280°C. The full-scan acquisition mode was used, with data collection over the m/z range of 50–450 amu, at a rate of 1 scan/s. The experimental results were compared with the NIST database, and only compounds with probability values greater than 800 were retained. The retention indices of the compounds were calculated using C_7_–C_40_ n-alkanes (Sigma-Aldrich, Co.) under the same conditions. Semi-quantification of the aroma compounds was performed according to the following equation (Luo et al., 2008): C = A_c_ × C_is_/A_is_; where C is the relative concentration of VFCs, C_is_ is the final concentration of the internal standard in the sample, A_c_ is the peak area of VFCs, and A_is_ is the peak area of the internal standard.

### Statistical Analysis

Statistical analysis was performed using the Statistical Package for the Social Sciences (SPSS 20.0, IBM Co., Chicago, IL, United States). The data obtained were subjected to one-way analysis of variance (ANOVA), with the significance level defined as *P* < 0.05. Principal component analysis (PCA) and bidirectional orthogonal projections to latent structures (O2PLS) were performed to analyze and screen the main flavor compounds using SIMCA^®^ (version 14.1, Sartorius Stedim Data Analytics AB, Umeå, Sweden). The RDA of environmental factors and microbial community evolution during *SZR* fermentation was performed using Canoco (version 4.5, Biometris Plant Research International, Wageningen University, Wageningen, The Netherlands). Pearson’s correlation was calculated using R to calculate the beneficial or antagonistic relationships between the microbiota and major flavor substances (VIP > 1, *P* < 0.05), and the network was created using Gephi (version 0.9.2^5^). All experiments were completed in quintuplicate, and data are expressed as means ± standard deviations (SD) from the mean.

## Results and Discussion

### Physicochemical Analysis

The variations in the physicochemical properties of the *SZR* samples are shown in Table 1. The differences in a_w_, pH, NaCl, and TVB-N content can be used to compare the quality differences and reflect the microbial status of *SZR* in the three regions. a_w_ and pH values of the three samples were significantly different (*P* < 0.05) and ranged from 0.9404 to 0.970 and 3.68 to 4.14, respectively. The differences are mainly due to the variation in the relative humidity and local climate during the fermentation process (Kim and Park, 2014). All samples were high-acid fermented meat products. The a_w_ value of LB was the highest, and that of ZY was the lowest; the lower the a_w_ value, the more stable the microorganisms, the better the preservation of food, and the higher the quality (Laranjo et al., 2015). LB also had the lowest pH compared to the other samples due to its high a_w_ value. The change in pH can be attributed to the accumulation of organic acids such as lactic and acetic acids (Juaárez-Castelaán et al., 2019).

**TABLE 1:** Physicochemical properties of *SZR* from different regions.

Sample	a_w_	pH	NaCl(%)	TVB-N(mg/100 g)
ZY	0.9404 ± 0.002^c^	4.14 ± 0.15^a^	10.33 ± 0.01^c^	20.15 ± 0.01^a^
MT	0.9408 ± 0.003^b^	4.08 ± 0.44^a^	11.60 ± 0.19^a^	11.26 ± 0.02^c^
LB	0.9470 ± 0.001^a^	3.68 ± 0.13^b^	11.05 ± 0.55^b^	11.62 ± 0.02^b^

*Values are presented as the mean ± standard deviation of quintuplicate (n = 5). ^a–c^Different letters in the same column represent significant differences (P < 0.05).*

TVB-N is considered one of the indicators for evaluating the freshness of meat products (Zhang et al., 2016). By the action of enzymes and bacteria, proteins are broken down to produce ammonia and alkaline nitrogenous substances such as amines, which affect the nutritional value of meat products (Lu et al., 2017). However, higher levels of NaCl can inhibit the growth of TVB-N, thereby retarding spoilage (Wang et al., 2020). Due to the higher NaCl content of MT, its TVB-N value was the lowest compared to the other samples.

### Abundance and Diversity of Bacterial and Fungal Microorganisms

**FIGURE 1:**
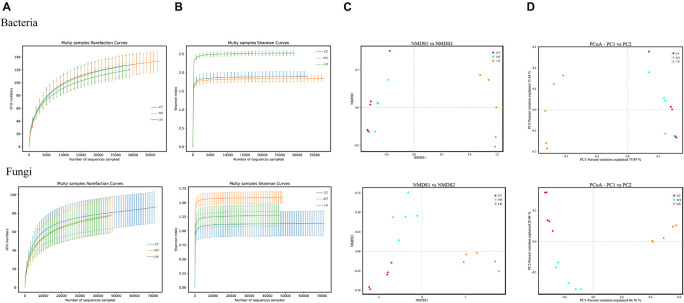
**(A)** Variations in microbial diversity and community structure of *SZR* in three different regions. rarefaction curves of fungi and bacteria for each sample. **(B)** Shannon index curves of fungi and bacteria for each sample. **(C,D)** NMDS and PCoA score plots of bacteria and fungi.

HTS was used to obtain 889,303 and 1,051,889 high-quality sequences from the 16S rRNA and ITS1 genes from the 15 *SZR* samples, respectively. The average numbers of effective sequences for fungi and bacteria were 70,126 and 59,287, respectively (Supplementary Table 2). In all samples fungal sequences significantly exceeded bacterial sequences; nonetheless, the total number of bacterial OTUs was much higher than that of fungi OTUs. Moreover, the OTU coverage of each sample was higher than 99.99%, indicating that almost all microorganisms were detected in the three samples. The rarefaction curves and Shannon index curves were close to the saturation plateau (Table 2), indicating that the sequencing data were sufficient for subsequent analysis (Figures 1A,B). The results of α-diversity showed that the LB samples had higher bacterial diversity than those from the other two regions, and MT had the highest fungal diversity. NMDS and PCoA, based on the binary Jaccard coefficient, were used to analyze the variability and similarity of the microbial population structure of *SZR* in different regions (Figures 1C,D). The results revealed differences in fungal community composition in the three regions but similar bacterial community composition in ZY and MT.

**TABLE 2:** Richness and diversity of bacteria and fungi at different processing stages.

Sample	ACE		Chao1		Simpson		Shannon	
	fungi	bacteria	fungi	bacteria	fungi	bacteria	fungi	bacteria
ZY	104.58 ± 18.79^a^	147.91 ± 29.08^a^	100.91 ± 26.81^a^	149.24 ± 28.13^a^	0.51 ± 0.16^a^	0.24 ± 0.02^a^	1.13 ± 0.43^a^	1.90 ± 0.27^b^
MT	90.06 ± 37.02^a^	148.24 ± 31.51^a^	93.22 ± 39.86^a^	149.72 ± 29.94^a^	0.27 ± 0.04^b^	0.26 ± 0.02^a^	1.59 ± 0.21^a^	1.85 ± 0.17^c^
LB	104.48 ± 26.46^a^	139.33 ± 22.36^a^	89.74 ± 31.32^a^	145.11 ± 15.66^a^	0.52 ± 0.12^a^	0.13 ± 0.01^b^	1.28 ± 0.37^a^	2.53 ± 0.11^a^

*Values are presented as the mean ± standard deviation of quintuplicate (n = 5). ^a–c^Different letters in the same column represent significant differences (P < 0.05).*

### Bacterial and Fungal Communities of *Suan zuo rou*

**FIGURE 2:**
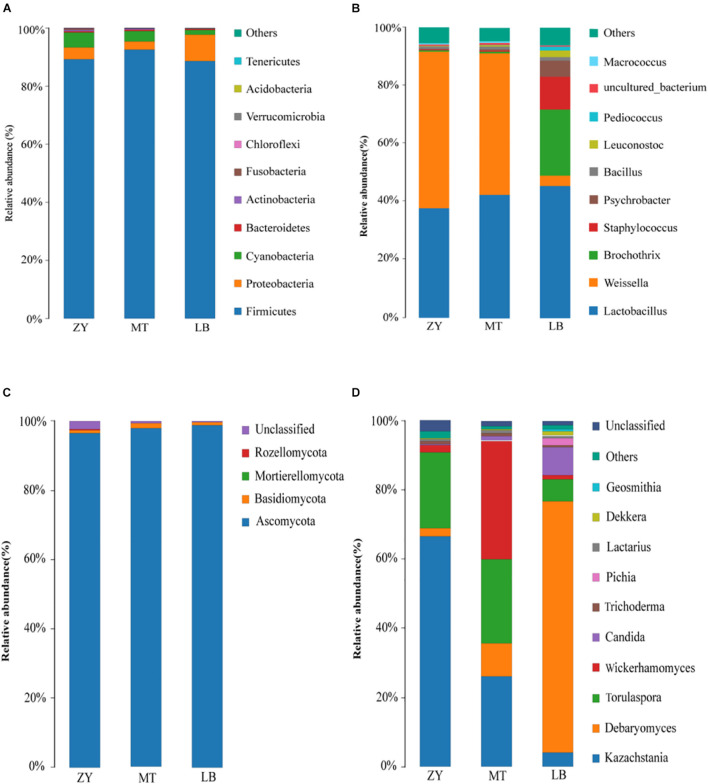
Relative abundance of bacteria at the phylum **(A)** and genus **(B)** level and fungi at phylum **(C)** and genus **(D)** of *SZR* from different regions.

Next, the sequencing data of fungi and bacteria were classified at both the phylum and genus levels to investigate the community structure in depth (Figures 2A–D). At the phylum level, 10 bacterial phyla and 4 fungal phyla were identified in the 15 *SZR* samples (Top 10 relative abundance). Among the bacterial communities, *Firmicutes* and *Proteobacteria* represented more than 90% of each sample sequence, followed by *Cyanobacteria*, *Bacteroidetes*, *Actinobacteria*, *Fusobacteria*, *Chloroflexi*, *Verrucomicrobia*, *Acidobacteria*, and *Tenericutes* (Figure 2A). *Firmicutes* dominated in all samples with 89.26%, 92.55%, and 88.61% abundance in ZY, MT, and LB, respectively, whereas the abundances of *Proteobacteria* were 4.05%, 2.81%, and 9.10% in ZY, MT, and LB, respectively (Figure 2A). Hu et al. (2020) reported that *Firmicutes* and *Proteobacteria* were also the dominant bacteria in traditional dry sausages from Northeast China. With respect to fungi, *Ascomycota* represented more than 96% of each sample sequence, followed by *Basidiomycota*, *Mortierelomycota*, and *Rozellomycota* (Figure 2C). The abundances of *Ascomycota* were 96.53%, 98.15%, and 98.86% in ZY, MT, and LB, respectively, whereas *Rozellomycota* was only detected in ZY and LB with an abundance of 0.01% and 0.0001% (Figure 2C), respectively.

At the genus level, 114 bacterial genera were identified in the 15 *SZR* samples. *Lactobacillus*, *Weissella*, *Brochothrix*, *Staphylococcus*, *Psychrobacter*, *Bacillus*, *Leuconostoc*, and *Pediococcus* were the 10 core genera (Top 10 relative abundance), as shown in Figure 2B. The highest abundance of *Lactobacillus* (44.95%) was found in LB, followed by MT (41.11%) and ZY (35.34%). *Lactobacillus* can release cytoplasmic enzymes and convert substrates from food matrix into aroma compounds of fermented meat products (Smid and Kleerebezem, 2014), and it has been reported to comprise the vast majority of the bacterial community of fermented meat products, for example, fermented llama meat sausages (Fontana et al., 2016) and chorizo de Leoìn (Quijada et al., 2018). *Staphylococcus* was detected at a much higher relative abundance in LB (11.15%) than MT (0.33%) and ZY (0.22%), which was consistent with the result of previous study (Lv et al., 2020). *Staphylococcus* is of interest because it have the ability to improve flavor mainly through the metabolism of proteins and lipids (Fonseca et al., 2013). Beneficial bacteria from the genera *Weissella*, *Bacillus*, *Leuconostoc*, and *Pediococcus* were also present in all *SZR* samples in different proportions. In addition, *Brochothrix*, which grows in aerobic or anaerobic environments, and is associated with meat spoilage, was found in LB with an abundance of 22.39% (Odeyemi et al., 2020). This high level of *Brochothrix* in LB may be caused by the fresh raw material and unacceptable hygienic conditions (Casaburi et al., 2014); therefore, care should be taken to detect and inhibit its growth in *SZR* production in the future.

A total of 57 fungal genera were identified in all samples, of which *Kazachstania*, *Debaryomyces*, *Torulaspora*, *Wickerhamomyces*, *Candida*, *Trichoderma*, *Pichia*, *Lactarius*, *Dekkera*, and *Geosmithia* were the most abundant (Figure 2D). *Kazachstania* was the major genus in ZY (66.70%), MT (26.14%), and LB (4.16%). In some fermented foods, VFCs, such as ethyl tetradecanoate, are produced by *Kazachstania* during fermentation, and play an important role as a flavoring agent (Tominaga, 2004; Kong et al., 2014). In addition, *Kazachstania* can play a vital part in the regulation of yeast communities, and has been detected in many traditional fermented foods, such as sourdough bread (Lhomme et al., 2015), camembert-type cheese (Bai et al., 2010), and wine (Sun and Liu, 2014). In contrast, *Debaryomyces* was the most abundant in LB (72.65%) and the least abundant in ZY (2.34%). *Debaryomyces* has been widely used as an auxiliary starter, and is a beneficial fungus in Panxian ham (Mu et al., 2019) as well as being important in the fermentation and maturation of sausage (Dalton et al., 1984). Furthermore, Fatichenti et al. (1983) reported that *Debaryomyces* also inhibits putrefactive bacteria. *Wickerhamomyces* has also been reported to show excellent antibacterial activity and antioxidant properties in meat products (Xu et al., 2019), and had the highest content in MT (34.22%), ZY (2.00%), and LB (1.04%). The significant differences in microbial community composition among *SZR* obtained from three different regions could be caused by factors such as raw materials, different ingredients, production environment, fermentation temperature, and relative humidity.

### Flavor Compounds Analysis

**FIGURE 3:**
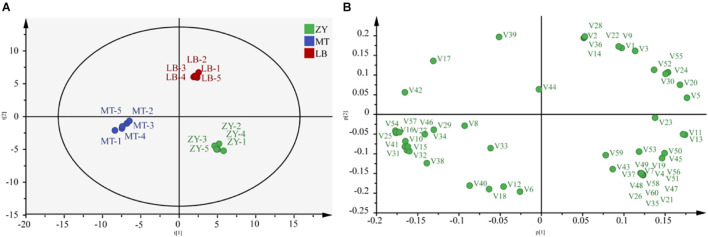
**(A)** Principal component analysis (PCA) and load diagram **(B)** results for flavor metabolites contents in *SZR* samples.

HS-SPME-GC-MS was used to detect VFCs in the *SZR* samples. In total, 60 VFCs were detected, consisting of 21 alcohols, 17 acids, 8 esters, 5 ketones, 3 aldehydes, 3 phenolic compounds, and 4 others (Supplementary Table 3). PCA showed that the differences between PC1 and PC2 were 90.2% and 5.7%, respectively (Figures 3A,B). There was a clear separation among ZY, MT, and LB, indicating differences in the flavor of the *SZR* from the three regions.

**FIGURE 4:**
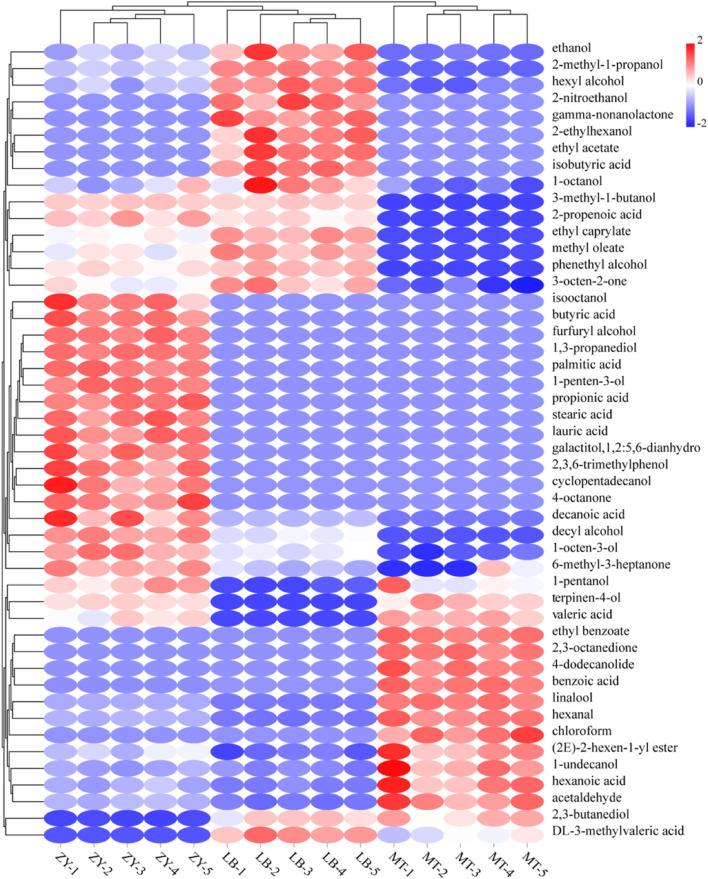
Heat map of changes in differential compounds of *SZR* from three different regions.

To better explore the VFCs that lead to the differences between groups, we performed O2PLS discriminant analysis to explore differences in the abundance of VFCs in these samples. Forty-eight types of VFCs were found to be significantly different across samples (VIP > 1, *P* < 0.05). A heat map was generated to investigate the relative quantification of the filtered differential VFCs and their relationship with the characteristics of *SZR* samples from three different regions (Figure 4).

Aldehydes are mostly derived from the oxidation of unsaturated fatty acids, while minimal quantities are also generated by the Maillard reaction. These compounds have low perception threshold values and a fruity odor, and are important VFCs in processed meat products (Lorenzo et al., 2014). The hexanal and non-anal detected in this study were also typical of Jinhua ham (Wang et al., 2021). Hexanal is obtained from the oxidation of n-6 fatty acids (oleic acid and arachidonic acid), which is also associated with grass flavors; however, excessive amounts of hexanal lead to putrefaction odors (Lorenzo et al., 2014). A significantly higher content of 1-non-anal hexanal, which imparts the fruity and green vegetable flavors of meat products, was detected in MT than in the other samples (Ramírez and Cava, 2007).

Esters, which have a particularly fruity flavor, contribute significantly to the formation of flavor qualities in fermented meats and were the main VFC in all *SZR* samples (Sidira et al., 2016). They are usually derived from the esterification of short-chain acids with alcohols (Herranz et al., 2005). Ethyl acetate (fruit and wine aromas), ethyl lactate (fruit aroma), and ethyl caprylate (floral and fruit aromas) were all significantly abundant (*P* < 0.05), with some, such as ethyl acetate and ethyl caprylate, being particularly abundant in the LB sample compared with the other samples. Ethyl benzoate, which can produce pear, apple, and banana aromas that enhance complexity, was also found in LB, which may be attributed to the higher ethanol content during fermentation (Zhao et al., 2020).

Ketones can be produced by lipid autoxidation and microbial metabolism, and are a source of animal flavor and vegetable fat flavor; in fermented meat, a high concentration of ketones can produce floral and spicy flavors (Sanchez-Pena et al., 2005). Methyl ketones are mainly derived from the decarboxylation of β-keto acid or the β-oxidation of saturated fatty acids, providing a certain fermented flavor in sour meat products (Chen et al., 2017). Acetone, 6-methyl-3-heptanone, and 3-octen-2-one were detected at significantly different levels in the three samples (*P* < 0.05), whereas 2,3 octanedione and 4-octanone were only found in the MT and ZY samples.

Volatile acids, which are mainly produced by hydrolysis of phospholipids and triglycerides, lipid oxidation, and the Maillard reaction, also contribute to the aroma and flavor characteristics of fermented meats (Ruiz et al., 2002). Short-chain acids (C < 6) have greater implications in VFCs in most samples because of their low perception threshold (Sidira et al., 2016). Acetic acid, propionic acid, isobutyric acid, butyric acid, n-pentanoic acid, and 3-methyl-pentanoic acid were the main short-chain acids found in this study. Long-chain (C14–C18) and medium-chain (C6–C12) acids are products of oxidative degradation of animal fats and are also important reactants formed by ester substances. Octanoic acid is a medium-chain (C6–C12) acid that was detected in the ZY and MT samples. It has an unpleasant smell at high concentrations and a fruity aroma after dilution, but its high threshold does not directly affect the flavor of ZY and MT, which can be inferred for certain other VFCs as well, such as methyl ketones and alcohols (Urbach, 1995).

Alcohols, another important component of *SZR* flavor, are closely related to lipid oxidation, amino acid metabolism, methyl ketone reduction, and microbial reproduction (Lin et al., 2018). Ethanol, 1-pentanol, 1-undecanol, cyclohexanol, 1-Octen-3-ol, linalool, 2,3-butanediol, 1-octanol, and phenethyl alcohol were significant in the *SZR* samples (*P* < 0.05). Phenyl alcohol was the highest in LB, which may be caused by carbohydrate decomposition and hydrate-induced microorganisms (such as yeast *Aspergillus oryzae*). 1-Octen-3-ol, which is oxidized by arachidonic acid and has a low odor threshold value, existed in all the samples but had the highest content in ZY, which had a marked odor of mushroom.

Apart from the above, linalool and terpene alcohol were detected in the 15 samples as components of anise and pepper, as well as *Artemisia argyi* (Summo et al., 2011). Although different manufacturers add a variety of spices, these compounds have a higher threshold value and contribute less to the overall flavor of *SZR* (Shahidi et al., 1986). Small amounts of trichloromethane, cyclopentane, and coconut aldehyde were also detected in MT, ZY, and LB, respectively, the first time these substances have been detected in *SZR*.

### Co-occurrence and Exclusion Analyses Revealed the Relationships Between Different Microbes

**FIGURE 5:**
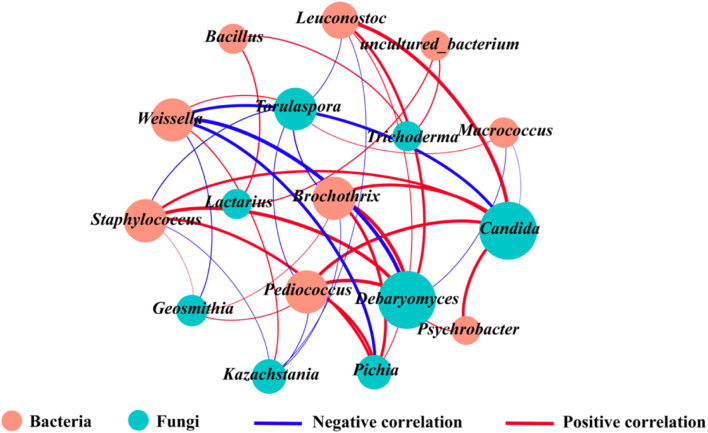
Association network diagram of bacteria and fungi. The orange and green circles refer to bacteria and fungi, respectively, and the red and blue lines refer to positive correlations (*r* > 0.7 and *P* < 0.05) and negative correlations (*r* < −0.7 and *P* < 0.05), respectively. (For interpretation of the references to color in this figure legend, the reader is referred to the web version of this article).

Microbial interactions are an important factor influencing the structure of microorganisms (Zhang et al., 2018). To understand the symbiotic or antagonistic relationships between different microbial genera, correlation co-occurrence networks between core bacteria and fungi were constructed using Pearson’s correlation coefficients and *P*-values (Figure 5). Correlation analysis between bacteria and fungi showed that *Brochothrix*, *Staphylococcus*, and *Pediococcus* were positively correlated with *Debaryomyces*, *Candida*, *Pichia*, and *Geosmithia* (| *r*| > 0.7, *P* < 0.05), whereas *Weissella* and *Macrococcus* were negatively correlated with *Debaryomyces* and *Candida* (| *r*| > 0.7, *P* < 0.05). Furthermore, *Staphylococcus*, *Brochothrix*, *Leuconostoc*, and *Pediococcus* together inhibited the growth of *Kazachstania* and *Torulaspora* (| *r*| > 0.7, *P* < 0.05), whereas *Weissella* was positively correlated with *Kazachstania* and *Torulaspora* (| *r*| > 0.7, *P* < 0.05). In addition, *Lactarius* was positively correlated with *Bacillus* and *uncultured_bacterium* (| *r*| > 0.7, *P* < 0.05). Differences among regions were mainly manifested in the relative abundance of *Weissella*, *Brochothrix*, *Staphylococcus*, *uncultured_bacterium*, *Debaryomyces*, *Kazachstania*, and *Torulaspora*, which are the dominant and functional genera known to enhance the aroma profile of fermented foods (Maturano et al., 2015). These findings were consistent with the HTS and microbial co-occurrence network results in our study; however, several microbial genera with high relative abundance, such as *Kazachstania* and *Lactobacillus*, were unexpectedly eliminated from the microbial correlation network.

### Correlations Between Microorganisms and Physiochemical Properties

**FIGURE 6:**
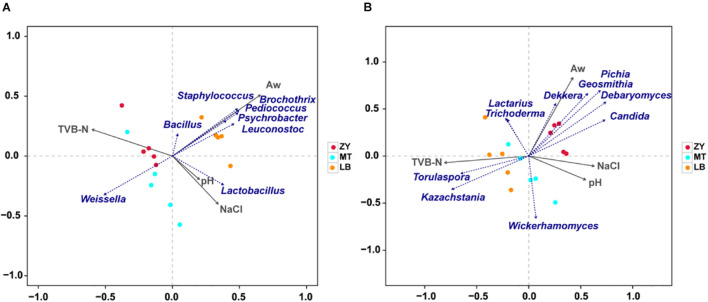
**(A)** RDA of microorganisms (Top 10 of OTU) and physiochemical properties. Correlation between physiochemical properties and fungal genera. **(B)** Correlations between physiochemical properties and bacterial genera.

Physicochemical properties of *SZR* were mainly determined by the production process and fermentation time, which had an obvious influence on the microbial community succession in the food microecological environment (Jung et al., 2014). The correlation between the physicochemical properties and microflora of *SZR* was identified by RDA (Figure 6), which showed that the microbial community structure was affected by pH, a_*w*_, NaCl, and TVB-N. In terms of bacteria communities, most of the dominant bacterium were positively related with a_*w*_, with *Staphylococcus*, *Psychrobacter*, *Bacillus*, *Leuconostoc*, *Pediococcus*, and *Macrococcus* showing the strongest positive correlation. Stavropoulou et al. (2018) also reported a significant effect of pH on the community structure of *Staphylococcus* during meat fermentation, and our finding that *Lactobacillus* showed a positive correlation with pH and a negative correlation with TVB-N is consistent with the findings of Zhang et al. (2018), who noticed that *Lactobacillus*, as a facultative anaerobe, has a significant influence on TVB-N (Figure 6A).

For fungi, the contribution of a_*w*_ to microbial community structure was 97.38%; therefore, it was considered that a_*w*_ was the main environmental factor that drove the variation in the fungus community in *SZR* (Figure 6B). *Dekkera* was positively correlated with a_*w*_, converts hydroxycinnamic acids to volatile phenols, which lead to meat spoilage (Lima et al., 2020). Given that the spoilage of meat products is known to depend on the amount of a_*w*_, these results indicate that the effect of a_*w*_ on meat spoilage could be linked to the growth and reproduction of *Dekkera* and other fungi (Oelofse et al., 2016). In terms of other fungal genera, *Torulaspora* and *Kazachstania* had a significantly positive correlation with TVB-N, whereas *Lactarius* and *Trichoderma* were negatively correlated with pH and NaCl, indicating that the concentration of NaCl had a greater effect on *Lactarius* and *Trichoderma* than *Dekkera*.

### Correlations Between Microorganisms and Volatile Flavor Compounds

**FIGURE 7:**
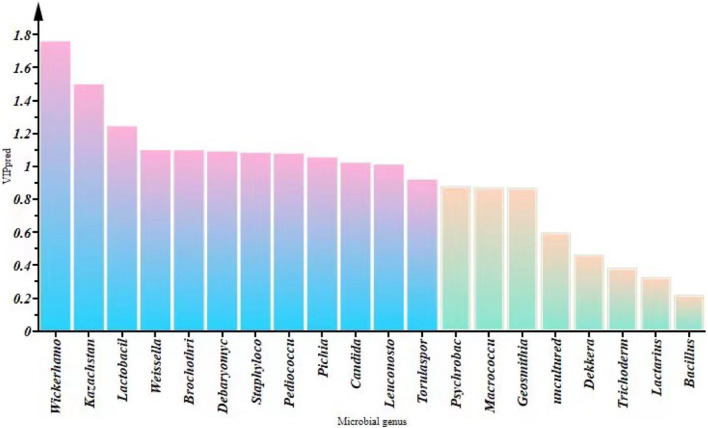
Changes in VIP (pred) values of the top 10 fungi and bacteria by relative abundance at the genus level.

We constructed an O2PLS model for VFCs and microorganisms in *SZR* samples from different regions (Supplementary Figure 1). The R^2^ and Q^2^ values of the O2PLS model were 0.983 and 0.891, respectively, which indicate that the O2PLS model has excellent interpretation rate and predictive capability (Triba et al., 2015). The VIP(pred) vector (calculation of variable importance for the projection) was applied to measure the intensity and explanatory ability of the influence of microorganisms on the formation of VFCs (Figure 7). A total of 12 microorganisms [VIP(pred) > 1.0], comprising 7 bacteria [VIP(pred):1.242–1.010] and 5 fungi [VIP(pred):1.758–1.021], had an influence on VFCs, of which *Wickerhamomyces*, *Kazachstania*, *Lactobacillus*, *Weissella*, *Brochothrix*, *Debaryomyces*, *Staphylococcus*, *Pediococcus*, *Pichia*, *Candida*, *Leuconostoc*, and *Torulaspora* were the primary influencers.

**FIGURE 8:**
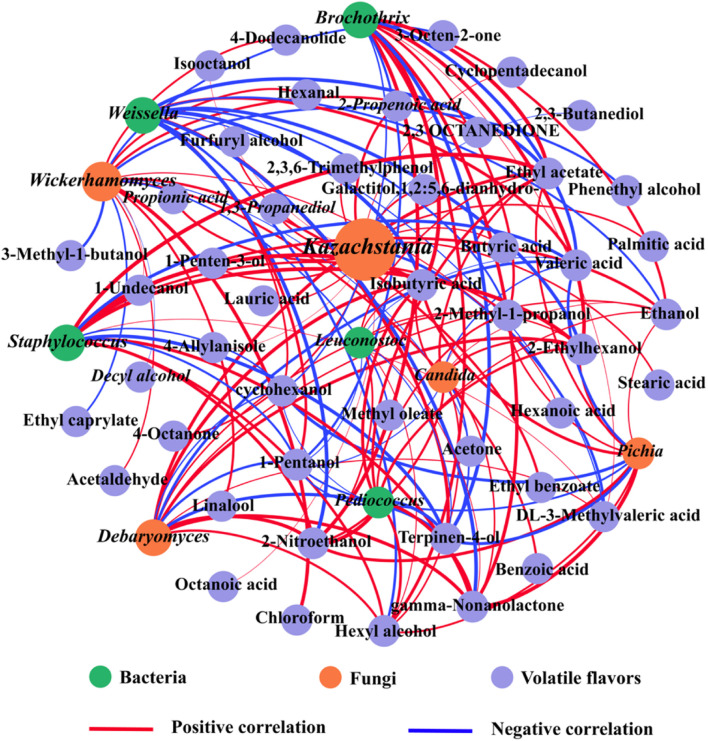
Correlation analyses between microorganisms [VIP(pred) > 1.0] and volatile flavor compounds. Statistically significant (*P* < 0.05) Pearson’s correlation coefficient (| ρ| > 0.8) indicates the robust correlations. The green, orange and purple circles refer to bacteria, fungi and volatile flavors, respectively, and the red and blue lines refer to positive correlations (| *r*| > 0.8 and *P* < 0.05) and negative correlations (| *r*| < −0.8 and *P* < 0.05), respectively. The size of nodes indicates the degree of connections. The thickness of each connection (edge) between two nodes is proportional to the value of Pearson’s correlation coefficient. (For interpretation of the references to color in this figure legend, the reader is referred to the web version of this article).

The interaction of VFCs with microorganisms was evaluated using Pearson’s correlation coefficient which showed that different microorganisms exerted different effects on VFCs (Figure 8). Based on Pearson’s correlation, a total of 5 bacterial and 5 fungal species were screened to be highly correlated with VFCs (| *r*| > 0.8, *P* < 0.05), namely, *Kazachstania*, *Debaryomyces*, *Wickerhamomyces*, *Candida*, *Pichia*, *Weissella*, *Brochothrix*, *Staphylococcus*, *Leuconostoc*, and *Pediococcus*. *Staphylococcus* plays a crucial role in the formation of the final flavor quality of fermented meat products (Iacumin et al., 2020), and was positively correlated with 8 volatile compounds, namely ethyl acetate, 2-ethylhexanol, isobutyric acid, gamma-non-anolactone, dL-3-methylvaleric acid, ethanol, hexyl alcohol, and 2-methyl-1-propanol, but negatively associated with terpinen-4-ol and valeric acid (| *r*| > 0.8, *P* < 0.05). This correlation might be related to the involvement of free amino acids produced by *Staphylococcus* owing to the hydrolysis of proteins, which can contribute to the formation of VFCs (Cruxen et al., 2017). In addition, LAB (including *Weissella* and *Leuconostoc*), which are probiotics that have beneficial effects on human health, were related to valeric acid, 1-pentanol, isobutyric acid, 2-ethylhexanol, and hexyl alcohol. They are also widely used as starters in many fermentation processes and might play a pivotal role in flavor development (Göğüş et al., 2004; Leroy et al., 2006). *Leuconostoc* can increase the content of some metabolites such as acids and alcohols (Galle et al., 2010; Xu et al., 2017), so it is not surprising that it is positively correlated with isobutyric acid, ethyl acetate, 2-ethylhexanol, hexyl alcohol, and 2-nitroethanol. In addition, *Pediococcus* was related to ethyl acetate and terpinen-4-ol. These have been widely used in the production of fermented foods because of their role in increasing the content of organic acids, short-chain fatty acids, and esters during food fermentation (Xu et al., 2021), which is consistent with our results.

With regards to fungi, *Kazachstania*, *Debaryomyces*, *Wickerhamomyces*, *Pichia*, and *Candida* are particularly important in fermented meat products and are the main microorganisms in alcohol and glycerol fermentation (Ai et al., 2019). The main VFCs of *SZR* are derived from the breakdown of proteins by yeasts and the conversion of amino acids (Engels et al., 1997). Ethanol, 2-nitroethanol, 2-methyl-1-propanol, hexyl alcohol, 1-pentanol, cyclohexanol, terpinen-4-ol and 2-ethylhexanol showed positive correlation with *Debaryomyces*, *Pichia*, and *Candida*, whereas furfuryl alcohol and cyclopentadecanol were only correlated with *Kazachstania*. Most yeasts were positively correlated with alcohols and esters, and *Debaryomyces* and *Picha* promoted the production of higher alcohols, acetate, and fatty acid esters. *Wickerhamomyces* can promote the Strecker degradation pathway, thereby producing aldehydes that increase the flavor of *SZR* (Bolumar et al., 2003), which is supported by the findings in this study that *Wickerhamomyces* was positively correlated with hexanal and acetaldehyde. Overall, *Weissella*, *Staphylococcus*, *Leuconostoc*, and *Pediococcus* bacteria, and *Kazachstania*, *Debaryomyces*, *Wickerhamomyces*, *Pichia*, and *Candida* fungi were the main sources of VFCs.

## Conclusion

In this study, we investigated the differences between the microbial communities, VFCs, and physicochemical properties of traditional *SZR* from three different regions of Guizhou province, China. Our findings revealed that the abundance of *Brochothrix* and *Staphylococcus* was significantly higher in LB samples than in ZY and MT samples, whereas that of *Wickerhamomydes* was markedly higher in the MT samples than in ZY and LB samples. Alcohols, acids, and esters were the main VFC found in all the samples. There were significant correlations between 48 volatile compounds and 10 core genera, namely *Wickerhamomyces*, *Kazachstania*, *Lactobacillus*, *Weissella*, *Brochothrix*, *Debaryomyces*, *Staphylococcus*, *Pediococcus*, *Pichia*, *Candida*, and *Leuconostoc*. The demonstration of the relationship among microbial communities, VFCs, and physicochemical properties provides a strong basis for further investigation of the microbial ecological impact of traditional fermented meat products. Future studies should use multi-omics approaches such as metagenomics, metaproteomics, and metatranscriptomics to study the fermentation mechanisms and metabolic pathways of *SZR* in depth, providing a scientific reference for the industrial production of traditional *SZR* in Guizhou province, China.

## Data Availability Statement

The datasets presented in this study can be found in online repositories. The names of the repository/repositories and accession number(s) can be found below: https://www.ncbi.nlm.nih.gov and SRP328727.

## Author Contributions

HW and WS contributed the experimental design, performed the statistical analysis, and wrote the manuscript. HW, WS, CZ, and YM contributed to manuscript revision, read, and approved the submitted version. All authors contributed to the article and approved the submitted version.

## Conflict of Interest

The authors declare that the research was conducted in the absence of any commercial or financial relationships that could be construed as a potential conflict of interest.

## Publisher’s Note

All claims expressed in this article are solely those of the authors and do not necessarily represent those of their affiliated organizations, or those of the publisher, the editors and the reviewers. Any product that may be evaluated in this article, or claim that may be made by its manufacturer, is not guaranteed or endorsed by the publisher.

## Footnotes

1
http://www.drive5.com/usearch/

2
https://www.arb-silva.de/

3
https://www.r-project.org/

4
https://gephi.org/

5
https://gephi.org/
