# Supplementary material for: Correlation Between Microbial Diversity and Volatile Flavor Compounds of Suan zuo rou, a Fermented Meat Product From Guizhou, China
Source: Front Microbiol. 2021 Oct 20;12:736525. doi: 10.3389/fmicb.2021.736525 (PMC8564356; doi:10.3389/fmicb.2021.736525)
Supplement: Supplementary file 1 [file Data_Sheet_1.docx]

Supplementary Material

# Supplementary Tables

**Supplementary Table 1** The main ingredients and processing conditions of sour meats in this study.

| Samples | Raw materials | Ingredients | Main seasonings | Processing season | Ripening temperature | Ripening time |
| --- | --- | --- | --- | --- | --- | --- |
| ZY | pork belly | rice flour | salt, Chinese pepper | Summer | ≤ 30 ºC | about 60 days |
| MT | pork belly | rice flour | salt | Summer | ≤ 30 ºC | about 45 days |
| LB | pork belly | rice flour | salt, Chinese pepper | Autumn | ≤ 20 ºC | about 30 days |

| Sample ID | Bacteria | | | |  | Fungi | | | |
| --- | --- | --- | --- | --- | --- | --- | --- | --- | --- |
|  | OTU | Effective Tags | Effective(%) | Coverage |  | OTU | Effective Tags | Effective(%) | Coverage |
| ZY-1 | 157 | 46156 | 100 | 0.9996 |  | 120 | 73627 | 91.89 | 0.9999 |
| ZY-2 | 170 | 57070 | 100 | 0.9997 |  | 118 | 74197 | 92.51 | 0.9999 |
| ZY-3 | 171 | 58468 | 100 | 0.9998 |  | 106 | 75283 | 94.38 | 0.9999 |
| ZY-4 | 105 | 72460 | 90.56 | 0.9998 |  | 46 | 79412 | 99.56 | 0.9998 |
| ZY-5 | 108 | 72053 | 90.12 | 0.9998 |  | 53 | 79317 | 99.58 | 0.9998 |
| MT-1 | 167 | 54953 | 100 | 0.9996 |  | 115 | 73141 | 94.13 | 0.9998 |
| MT-2 | 168 | 55238 | 100 | 0.9998 |  | 115 | 71858 | 93.67 | 0.9999 |
| MT-3 | 165 | 41596 | 100 | 0.9996 |  | 113 | 50303 | 93.86 | 0.9998 |
| MT-4 | 105 | 70934 | 88.58 | 0.9999 |  | 35 | 79318 | 99.39 | 0.9999 |
| MT-5 | 104 | 70262 | 87.44 | 0.9998 |  | 43 | 77535 | 99.34 | 0.999 |
| LB-1 | 144 | 32988 | 100 | 0.9994 |  | 112 | 52221 | 94.9 | 0.9998 |
| LB-2 | 146 | 32749 | 100 | 0.9993 |  | 110 | 57781 | 95.41 | 0.9998 |
| LB-3 | 144 | 41669 | 100 | 0.9993 |  | 107 | 47339 | 95.43 | 0.9997 |
| LB-4 | 107 | 65457 | 81.75 | 0.9998 |  | 43 | 79269 | 99.39 | 0.9999 |
| LB-5 | 102 | 63839 | 79.52 | 0.9997 |  | 39 | 79437 | 99.39 | 0.9998 |

**Supplementary Table 2** Statistics of sequencing and bioinformatics analysis

**Supplementary Table 3** Contents of volatile flavor compounds of *Suan zuo rou* from different regions

| **No.** | **Compound** | **Retention time（min）** | **RI** | **Content (μg/100g)** | | | **Odour description（s）** |
| --- | --- | --- | --- | --- | --- | --- | --- |
|  | **Alcohols** |  |  | **ZY** | **MT** | **LB** |  |
| V1 | Ethanol | 3.57 | 927 | 2.43±0.26^b^ | 1.49±0.09^c^ | 4.62±0.58^a^ | Wine |
| V2 | 2-Nitroethanol | 4.37 | 1038 | n.d. | n.d. | 0.53±0.09^a^ | n.d. |
| V3 | 2-Methyl-1-propanol | 7.84 | 1111 | 0.20±0.02^b^ | n.d. | 0.53±0.01^a^ | Malty |
| V4 | 1-Penten-3-ol | 9.57 | 1195 | 0.13±0.01^a^ | n.d. | n.d. | Green vegetable, fruity |
| V5 | 3-Methyl-1-butanol | 10.96 | 1223 | 3.33±0.09^a^ | n.d. | 3.27±0.08^a^ | Whiskey, malt, burnt |
| V6 | 1-Pentanol | 12.25 | 1146 | 1.84±0.13^a^ | 1.66±0.27^a^ | 0.86±0.04^b^ | Fruity, alcoholic, green |
| V7 | Isobutanol | 14.32 | 1115 | 0.31±0.07^a^ | n.d. | n.d. | Wine, solvent, bitter |
| V8 | 1-Tridecanol | 14.33 | 1058 | n.d. | 2.04±2.75^a^ | n.d. | Musty |
| V9 | Hexyl alcohol | 15.20 | 1364 | 0.63±0.08^b^ | 0.40±0.07^c^ | 1.24±0.06^a^ | Green, fruity, apple-skin and oily |
| V10 | 1-Undecanol | 17.21 | 1205 | 0.64±0.04^b^ | 1.32±0.20^a^ | 0.55±0.04^c^ | Oily clean waxy |
| V11 | Decyl alcohol | 17.22 | 1157 | 1.07±0.07^a^ | n.d. | 0.58±0.06^b^ | Orange, oil |

Contents of volatile flavor components of *Suan zuo rou* from different regions (continued).

| V12 | Cyclohexanol | 17.83 | 1392 | 1.32±0.06^a^ | 1.31±0.06^a^ | 0.97±0.10^b^ | n.d. |
| --- | --- | --- | --- | --- | --- | --- | --- |
| V13 | 1-Octen-3-ol | 18.35 | 1451 | 6.17±0.43^a^ | 2.30±0.34^b^ | 4.41±0.21^c^ | Mushroom-like |
| V14 | 2-Ethylhexanol | 18.83 | 1384 | n.d. | n.d. | 0.22±0.05^a^ | Rose, green |
| V15 | Linalool | 20.28 | 1556 | 3.36±0.03^b^ | 15.37±0.50^a^ | 0.47±0.05^c^ | Flower, citrus, orange |
| V16 | 2,3-Butanediol | 21.08 | 1590 | 0.35±0.06^b^ | 3.52±0.46^a^ | 3.25±0.43^a^ | Cream |
| V17 | Terpinen-4-ol | 21.49 | 1535 | 0.14±0.00^a^ | 0.15±0.02^a^ | n.d. | Turpentine, nutmeg, must |
| V18 | 1-Octanol | 22.01 | 1745 | 1.12±0.09^b^ | 0.97±0.04^c^ | 1.31±0.12^a^ | Chemical, metal, burnt |
| V19 | Furfuryl alcohol | 22.62 | 1199 | 1.38±0.07^a^ | n.d. | n.d. | Burn |
| V20 | Phenethyl alcohol | 28.44 | 1193 | 4.52±0.24^b^ | 0.26±0.02^c^ | 5.32±0.27^a^ | Floral |
| V21 | 1,3-Propanediol | 33.62 | 1521 | 1.97±0.08^a^ | n.d. | n.d. | Emollients |
|  | **Aldehydes** |  |  |  |  |  |  |
| V22 | Hexanal | 6.95 | 1053 | 2.55±0.06^b^ | 6.53±0.35^a^ | 1.35±0.09^c^ | Grass, tallow, fat |
| V23 | Acetaldehyde | 6.99 | 712 | 3.05±0.13^b^ | 6.39±0.71^a^ | 1.96±0.14^c^ | Cabbage |

Contents of volatile flavor components of *Suan zuo rou* from different regions (continued)

| V24 | 1-Nonanal | 16.27 | 1381 | 1.03±1.00^a^ | 1.64±1.61^a^ | 0.10±0.01^b^ | Fat, citrus, green |
| --- | --- | --- | --- | --- | --- | --- | --- |
|  | **Esters** |  |  |  |  |  |  |
| V25 | Ethyl acetate | 3.10 | 912 | n.d. | n.d. | 0.48±0.09^a^ | Solvent, pineapple, fruity |
| V26 | Ethyl lactate | 14.98 | 1351 | 1.10±0.48^a^ | 0.31±0.02^b^ | 0.87±0.14^a^ | Fruity |
| V27 | Ethyl caprylate | 17.38 | 1428 | 0.22±0.02^b^ | n.d. | 0.32±0.02^a^ | Spice |
| V28 | Ethyl benzoate | 23.09 | 1645 | n.d. | 5.76±0.28^a^ | n.d. | Fruity |
| V29 | 4-Dodecanolide | 30.77 | 1372 | n.d. | 0.08±0.01^a^ | n.d. | Fruity |
| V30 | gamma-Nonanolactone | 30.77 | 1355 | n.d. | n.d. | 0.69±0.09^a^ | Coconut |
| V31 | Methyl oleate | 38.63 | 1154 | 0.44±0.06^b^ | n.d. | 0.65±0.06^a^ | n.d. |
| V32 | (2E)-2-hexen-1-yl ester | 22.94 | 837 | 4.66±0.41^b^ | 6.87±0.77^a^ | 3.04±0.33^c^ | n.d. |
|  | **Acids** |  |  |  |  |  |  |
| V33 | Acetic acid glacial | 17.97 | 1453 | 8.61±0.36^a^ | 10.05±0.74^b^ | 8.49±0.60^a^ | Sour |
| V34 | Propionic acid | 20.16 | 1531 | 1.17±0.09^a^ | n.d. | n.d. | Acidic |
| V35 | Isobutyric acid | 20.83 | 1554 | n.d. | n.d. | 0.82±0.09^a^ | Rancid, butter, cheese |
| V36 | Butyric acid | 22.27 | 1622 | 0.20±0.02^a^ | n.d. | n.d. | Rancid, cheese, sweat |

Contents of volatile flavor components of *Suan zuo rou* from different regions (continued)

| V37 | DL-3-Methylvaleric acid | 23.21 | 1293 | n.d. | 0.38±0.08^b^ | 0.66±0.07^a^ | Sour |
| --- | --- | --- | --- | --- | --- | --- | --- |
| V38 | Valeric acid | 24.76 | 1956 | 0.35±0.05^b^ | 0.44±0.03^a^ | n.d. | Sweat, acid, rancid |
| V39 | 2-Propenoic acid | 25.86 | 1231 | 1.08±0.11^a^ | n.d. | n.d. | n.d. |
| V40 | Hexanoic acid | 27.06 | 1861 | 12.83±1.30^b^ | 28.02±4.39^a^ | 9.25±0.66^c^ | Sweaty, cheesy, sharp |
| V41 | Heptanoic acid | 29.25 | 2168 | n.d. | 0.71±0.17^a^ | 0.36±0.07^b^ | Fresh, herbal |
| V42 | Octanoic acid | 31.34 | 1070 | 1.43±0.07^a^ | 1.08±0.21^b^ | 1.05±0.06^c^ | Sweat, cheese |
| V43 | Undecanoic acid | 31.71 | 1281 | n.d. | 0.79±0.67^a^ | n.d. | Waxy |
| V44 | Nonanoic acid | 33.33 | 2201 | 0.65±0.08^c^ | 0.97±0.51^b^ | 1.63±2.08^a^ | Green, fat |
| V45 | Decanoic acid | 35.24 | 2278 | 3.83±0.89^a^ | n.d. | 0.98±0.05^b^ | Rancid, fat |
| V46 | Benzoic acid | 38.01 | 1659 | n.d. | 20.41±1.16^a^ | 0.52±0.04^b^ | Urine |
| V47 | Lauric acid | 38.80 | 1567 | 0.46±0.05^a^ | n.d. | n.d. | Metal |
| V48 | Palmitic acid | 45.16 | 1970 | 4.31±0.26^a^ | n.d. | n.d. | Waxy |
| V49 | Stearic acid | 48.09 | 2752 | 2.09±0.22^a^ | n.d. | n.d. | Odorless mild fatty waxy |

Contents of volatile flavor components of *Suan zuo rou* from different regions (continued)

|  | **ketone** |  |  |  |  |  |  |
| --- | --- | --- | --- | --- | --- | --- | --- |
| V50 | Acetone | 14.08 | 1257 | 0.50±0.05^b^ | 0.27±0.05^c^ | 0.64±0.09^a^ | Solvent ethereal apple pear |
| V51 | 3-Heptanone, 6-methyl- | 14.95 | 1333 | 1.10±0.08^a^ | 0.40±0.39^c^ | 0.58±0.07^b^ | n.d. |
| V52 | 2,3 Octanedione | 14.98 | 1353 | n.d. | 2.90±0.17^a^ | n.d. | n.d. |
| V53 | 3-Octen-2-one | 17.63 | 1376 | 1.38±0.07^b^ | 0.83±0.12^c^ | 1.63±0.11^a^ | Nut, crushed bug |
| V54 | 4-Octanone | 20.29 | 1630 | 0.61±0.08^a^ | n.d. | n.d. | n.d. |
|  | **phenolic** |  |  |  |  |  |  |
| V55 | Phenol,2,4,5-trimethyl- | 30.21 | 867 | 0.37±0.06^a^ | n.d. | 0.18±0.03^b^ | n.d. |
| V56 | 2,3,6-Trimethylphenol | 30.26 | 1573 | 0.06±0.01^a^ | n.d. | n.d. | n.d. |
|  | **Else** |  |  |  |  |  |  |
| V57 | Chloroform | 5.88 | 1243 | n.d. | 0.05±0.01^a^ | n.d. | n.d. |
| V58 | Galactitol,1,2:5,6-dianhydro- | 20.15 | 1475 | 0.83±0.12^a^ | n.d. | n.d. | n.d. |
| V59 | 4-Allylanisole | 26.44 | 1645 | 0.62±0.59^b^ | n.d. | n.d. | anisic |
| V60 | Cyclopentadecanol | 32.65 | 1827 | 0.34±0.07^c^ | n.d. | n.d. | n.d. |

Values are presented as the mean ± standard deviation of quintuplicate(n=5).

^a-c^Different letters in the same row represent significant differences (*P*<0.05).

# Supplementary Figures


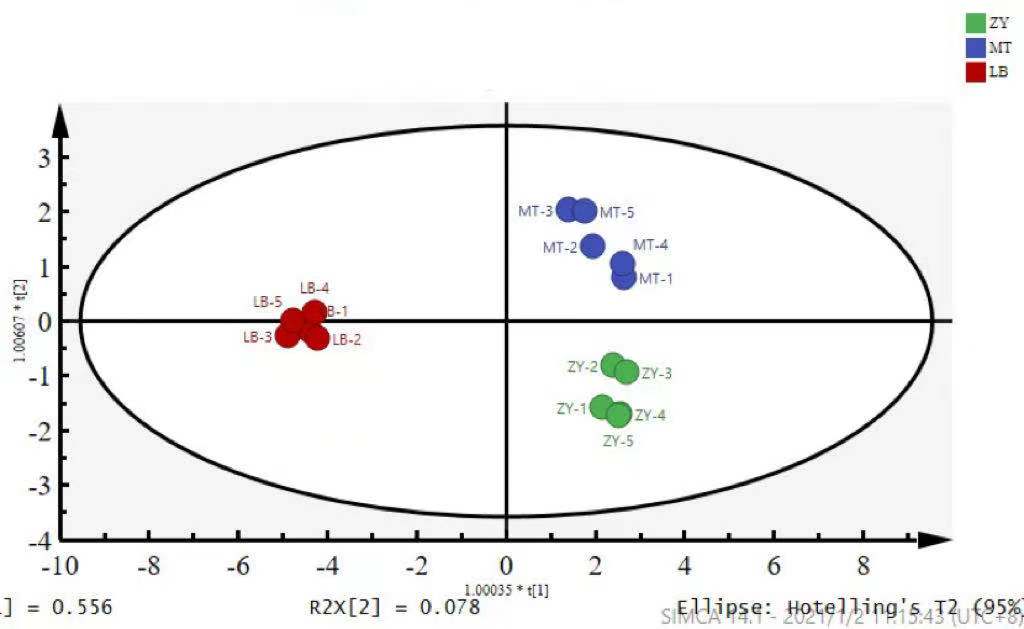


**Supplementary Figure 1.** Score plot of the two principal components after bidirectional orthogonal partial least squares (O2PLS) of the relative abundance of microbiota and volatile compounds in Suan zuo rou.
